# Mepolizumab as Possible Treatment for Allergic Bronchopulmonary Aspergillosis: A Review of Eight Cases

**DOI:** 10.7759/cureus.9684

**Published:** 2020-08-12

**Authors:** Amirseena Tolebeyan, Oranus Mohammadi, Zahra Vaezi, Afshin Amini

**Affiliations:** 1 Internal Medicine, St. Luke's Hospital, St. Louis, USA; 2 Internal Medicine, Aventura Hospital and Medical Center, Aventura, USA; 3 Internal Medicine, Zahedan University of Medical Sciences, Zahedan, IRN; 4 Internal Medicine, St. Luke's Hospital, Chesterfield, USA

**Keywords:** mepolizumab, allergic bronchopulmonary aspergillosis

## Abstract

Allergic bronchopulmonary aspergillosis (ABPA) is an eosinophilic pulmonary disorder caused by a hypersensitivity reaction to Aspergillus fumigatus that manifests with uncontrolled asthma, peripheral blood eosinophilia, and radiological findings, such as mucus plugging. Early diagnosis and proper treatment of ABPA are essential to prevent irreversible lung damage such as pulmonary fibrosis and bronchiectasis and improve the quality of life of patients. Beside inhaled medication for asthma, anti-inflammatory agents (i.e., systemic glucocorticoids) and antifungal agents are the mainstay treatment of ABPA. The goal of therapy using glucocorticoids and antifungal agents is to suppress the immune hyperreactivity to A. fumigatus and attenuate the fungal burden. Since the systemic glucocorticoid therapy may lead to serious adverse effects including osteoporosis, avascular necrosis, myopathy, cushingoid appearance, hypertension, insomnia, and increased risk of infection, a glucocorticoid-sparing agent could be considered. Mepolizumab is a humanized monoclonal antibody that binds to interleukin-5, which is the key mediator for eosinophil differentiation, activation, migration, and survival. We review eight cases of ABPA treated successfully with mepolizumab. Treatment with mepolizumab was not restricted to the total immunoglobulin E level, the limiting factor for omalizumab in ABPA. In addition, mepolizumab therapy improved forced expiratory volume in one second, radiological findings, and patient quality of life.

## Introduction and background

Allergic bronchopulmonary aspergillosis (ABPA) was first described in 1952 [1]. ABPA is an eosinophilic pulmonary disorder caused by a hypersensitivity reaction to Aspergillus fumigatus, which manifested with uncontrolled asthma, peripheral blood eosinophilia, and radiological findings such as mucus plugging [2]. The prevalence of ABPA in asthma is not apparent due to the absence of extensive community-based data. However, the pooled prevalence of ABPA in asthma was reported at 8.4% [3]. Genetic studies showed that HLA-DR2 HLA-DRB1*1501 and HLA-DRB1*1503 genotypes provide high relative risk, but the presence of HLA-DQ2 DQB1*0201 protects against the development of ABPA [4]. The International Society for Human and Animal Mycology working group for ABPA proposed new criteria for the diagnosis of ABPA as follows: a predisposing condition (asthma or cystic fibrosis [CF]; one must be present), necessary criteria (elevated total serum immunoglobulin [Ig] E > 1,000 IU/mL and a positive Aspergillus skin test or detectable IgE against A. fumigatus; both elevated total IgE and proved presence of Aspergillus must be existent), supportive criteria (eosinophilia > 500 cells/µL, precipitating serum antibodies to A. fumigatus and radiological pulmonary findings consistent with ABPA; at least two criteria must be present) [3]. Early diagnosis and proper treatment of ABPA are essential to prevent irreversible lung damage such as pulmonary fibrosis and bronchiectasis and improve the quality of life of patients. Beside inhaled medication for asthma, anti-inflammatory agents (i.e., systemic glucocorticoids) and antifungal agents are the mainstay treatment of ABPA. Therapy with glucocorticoids and antifungal agents suppresses the immune hyperreactivity to A. fumigatus and attenuates fungal burden [5]. Five stages have been described for ABPA in patients with asthma: acute (I), remission (II), exacerbation (III), steroid-dependent asthma (IV), and end-stage fibrotic (V) [4]. Systemic glucocorticoids such as prednisone with an initial dose of 0.5 mg/kg/day are considered the core of treatment of ABPA in the acute phase. Patients in remission are off systemic glucocorticoids. ABPA exacerbation is managed by a tapered glucocorticoid regimen; however, some patients are unable to taper off of glucocorticoids [6]. Antifungal therapy with itraconazole or voriconazole is considered for patients who are unable to taper systemic glucocorticoids [7]. Since the systemic glucocorticoid therapy may lead to serious adverse effects including osteoporosis, avascular necrosis, myopathy, cushingoid appearance, hypertension, insomnia, and increased risk of infection, a glucocorticoid sparing agent could be considered. Omalizumab, a humanized monoclonal antibody that recognizes and binds to the CH3 domain of IgE and can neutralize free IgE, eventually leads to inhibition of IgE allergic pathway without sensitizing mast cells and basophils. Omalizumab has shown to have beneficial effects in the treatment of ABPA [8,9]. Mepolizumab is a humanized monoclonal antibody that binds to interleukin-5 (IL-5), which is the key mediator for eosinophil differentiation, activation, migration, and survival. Mepolizumab reduces exacerbations and improves the quality of life in patients with severe eosinophilic asthma [10]. We review eight cases of ABPA treated with mepolizumab.

## Review

A systematic literature review using PubMed and Google Scholar databases was performed. We included studies in English published up to October 2019. A total of eight cases were identified.

Results

Tables 1 and 2 present the results of our review of eight cases [11-17]. All patients experienced uncontrolled asthma and were diagnosed with ABPA based on eosinophilia, proven the presence of A. fumigatus, characteristic imaging findings, elevated total IgE levels (except Soeda et al.; case 2), and eosinophilia [14].

**Table 1:** Patient characteristics F, female; M, male; ADL, activities of daily living; O_2_, oxygen; GCC, glucocorticoid; MAC: Mycobacterium avium complex; SE, side effects; PSL, prednisolone; ITCZ, itraconazole; NA, not available.

Author	Age (years)	Sex	Reason to start mepolizumab	Biologics	PSL-ITCZ	Time to improvement	Total follow-up period after mepolizumab
Altman et al. [11]	56	F	Loss of ADLs, GCC, and O_2_ dependent	Omalizumab, mepolizumab	-	6 months	6 months
Matsumoto et al. [12]	67	F	Frequent exacerbations	Mepolizumab	-	2 weeks	20 months
Oda et al. [13]	33	M	Frequent exacerbations, loss of ADLs	Mepolizumab	2.5 mg/day	8 weeks	8 months
Soeda et al. [14]	54	F	Patient refused GCC and antifungals	Mepolizumab	-	6 months	24 months
	66	F	Patient refused GCC due to previous SE	Mepolizumab	-	NA	21 months
Tsubouchi et al. [15]	65	F	MAC infection on GCC	Mepolizumab	5-200 mg/day	5 months	5 months
Terashima et al. [16]	64	F	Exacerbation on GCC off state, GCC SEs	Mepolizumab	-	4 weeks	4 weeks
Hirota et al. [17]	63	F	Radiological findings not improved	Omalizumab-mepolizumab	-	56 weeks	56 weeks

**Table 2:** Parameters changes after mepolizumab therapy Eos, eosinophil; N/C, not changed; FEV1, forced expiratory volume in the first second; NA, not available; FeNO, fractional exhaled nitric oxide; ACT score, asthma control test score.

Author	Eos/mm^3^	IgE IU/mL	FEV1	FeNO	ACT score	Radiological findings	Clinically
Altman et al. [11]	1,100→ Zero	1,730→ 298	40 mL	NA	NA	NA	Improved
Matsumoto et al. [12]	1,163→ 121 (after 2 weeks)	3,163→ 2,863 (after 2 weeks)	990 mL (after 12 months)	NA	NA	Improved (after 12 months)	Improved
Oda et al. [13]	6,370→ 64 (after 8 weeks)	182 (baseline)	800 mL (after 24 weeks)	230→ 105	5→ 25	Improved (after 32 weeks)	Improved
Soeda et al. [14]	1,365→73 (after 6 months)	2,145→ N/C	90 mL (after 6 months)	NA	21→ 25	Improved (after 6 months)	Improved
	1,856→ 32 (after 2 months)	162→ N/C	NA	NA	24→ N/C	Improved (after 4 months)	Improved
Tsubouchi et al. [15]	276→ 20 (after 3 months)	4,970→ 2,157 (after 3 months)	60 mL	67→ 39	NA	Improved (after 5 months)	Improved
Terashima et al. [16]	3,017→ 174	N/C	270 mL	NA	18→ 24	Improved (after 4 weeks)	Improved
Hirota et al. [17]	800→ Zero (after 56 weeks)	1,121→ 362 (after 56 weeks)	N/C	NA	NA	Improved (after 56 weeks)	Improved

CT scan of the lungs was performed for all patients. Mucoid impaction was the most common finding. Bronchiectasis was detected in five patients. Pulmonary infiltration, scattered nodules, bronchial wall thickening, and pulmonary fibrosis were the other findings on chest CT scan.

All patients were adults with the mean age of 58 years at the time of mepolizumab therapy. Subcutaneous mepolizumab 100 mg every four weeks was given to all patients. Seven patients were female and one was male. Two patients experienced frequent exacerbation while on systemic glucocorticoids, and one patient developed mycobacterium avium complex infection as the adverse effect of glucocorticoid therapy. One patient was treated with omalizumab and mepolizumab simultaneously. Omalizumab was switched to mepolizumab in one patient due to inadequate response. Two patients needed to continue low-dose glucocorticoid, but the clinicians did not try to taper glucocorticoid while the patients were on mepolizumab. The mean follow-up time was 12.25 ± 8 months. The mean eosinophil count before treatment with mepolizumab was 1,990 ± 1,800 mm^3^, and the eosinophil count in one patient was 276/mm^3^. In all patients, eosinophil count dropped significantly after treatment with mepolizumab, but IgE levels were reduced in only four patients. Forced expiratory volume in one second (FEV1) improved by a mean of 375 mL for all eight patients. All patients were clinically improved in a mean period of 4.8 ± 4 months, and radiological findings, including mucoid impaction, were resolved in seven patients (the report of radiological findings is not available in one patient). Asthma control test (ACT) score was reported in four patients: two patients had an ACT score <19, and the ACT score improved (reduced to 20 and 6) after treatment with mepolizumab in two other patients. 

Two patients had chronic rhinosinusitis with nasal polyps (CRSwNP) in addition to ABPA. In one patient (Matsumoto et al.), chronic sinusitis symptoms, including nasal discharge and nasal obstruction, improved two weeks after the initial mepolizumab administration, while CRSwNP in the other patient (Oda et al.) did not respond to mepolizumab therapy and the patient required endoscopic sinus surgery [12,13]. Among eight patients, no adverse effect of mepolizumab was reported.

Discussion

ABPA is a hypersensitivity reaction to A. fumigatus colonization in the lungs characterized by elevated total IgE, IgG, and IgE anti-A. fumigatus antibodies, eosinophilia, and various features of lung involvement, including pulmonary infiltrate, bronchiectasis, and pulmonary fibrosis [18].

ABPA is a chronic inflammatory disease that involves patients with asthma or CF [5]. The defective clearance function (of conidia) in the airways of patients with asthma or CF allows Aspergillus to grow and colonize. Both innate and adaptive immunity are involved in ABPA pathogenesis. Airway epithelium, dendritic cells, and macrophages recognize fungal products by two types of receptors: pattern recognition receptors (e.g., Toll-like receptors, nucleotide-binding oligomerization domain-like receptors, and C-type lectin receptors) and the complement receptor 3 pathway. Innate immune response releases chemokines and cytokines that activate the adaptive immune system. In a healthy individual, T helper (Th)1 CD4^+^ T cells response clears A. fumigatus from the airways. However, the immune response in ABPA is different and related to Th2 CD4^+^ T cells. In this pathway, chemokine (C-C motif) ligand (CCL)17 and CCL22 bind to C-C chemokine receptor type 4 on Th2 cells and activate Th2 cells. Therefore, T cells play a key regulatory role in the pathogenesis of ABPA, particularly Th2 cells. CD4^+^ cells produce IL-4, IL-5, IL-9, and IL-13, which induce the IgE immune response and promote eosinophilia. The intense inflammatory reaction with mast cell degranulation, presence of neutrophils and eosinophils, and IgE production cause the specific pathological characteristics of ABPA, such as bronchiectasis, mucus plugging, and eosinophilic pneumonia [5,18].

The goal of therapy in ABPA is to reduce IgE levels by 25% to 50%, which correlates with clinical and radiological improvement [19]. Systemic glucocorticoids are considered the treatment of choice in ABPA. Lower doses of glucocorticoids (e.g., prednisolone 0.5 mg/kg/day) are associated with a higher rate of relapses or glucocorticoid-dependent ABPA compared to a higher dose or prolonged duration of glucocorticoids (e.g., prednisolone 0.75 mg/kg/day). Nonetheless, even with high-dose glucocorticoid therapy, some patients are classified as glucocorticoid-dependent ABPA [3,20]. The anti-inflammatory effects of glucocorticoids are produced by inhibiting the recruitment of inflammatory cells, including eosinophils and T lymphocytes, through suppressing the production of chemotactic mediators and adhesion molecules [21].

Nevertheless, long-term systemic glucocorticoids provide a risk of serious adverse effects such as osteoporosis, avascular necrosis, myopathy, cushingoid appearance, hypertension, insomnia, and increased risk of infection [8]. To avoid the serious adverse effects of glucocorticoids, reduce the rate of exacerbation, and improve patient quality of life, a glucocorticoid-sparing agent is needed. Omalizumab is a humanized monoclonal antibody that recognizes and binds to the CH3 domain of IgE and deactivates free IgE, inhibiting the IgE allergic pathway and, therefore, preventing mast cells and basophils activation [9]. Omalizumab is an effective treatment for severe allergic asthma that can improve asthma symptoms and quality of life, and results in a significant reduction in the frequency of asthma exacerbation. A synthesis review of published literature showed that omalizumab improved fractional exhaled nitric oxide test scores (a biomarker of lung inflammation), symptoms, exacerbation rate, steroid use, and serum IgE levels in ABPA patients, and lung function was not significantly improved after omalizumab treatment [9]. Omalizumab dosing is restricted to body weight, and pretreatment total IgE level based on this formula: weight × IgE × 0.016 [22]. The maximum dose of omalizumab is 600 mg every two weeks. Consequently, omalizumab can be used in patients with a baseline total IgE in the range of 30-700 IU/mL. Total IgE levels greater than 1,000 IU/mL is an obligatory criterion for ABPA, and many patients with ABPA (even in the exacerbation phase) have high total IgE levels; subsequently, omalizumab therapy is limited in these patients [5].

Mature eosinophils are generated from CD34+ progenitors through the effect of IL-5, which are produced by Th2 cells. Mepolizumab is a monoclonal antibody that binds to IL-5 and prevents maturation of eosinophil in the bone marrow and reduces eosinophil numbers in airways [23]. Mepolizumab showed to be effective in severe eosinophilic asthma by reducing exacerbations and improving asthma control scores [10]. 

In the current study, we assess the response to mepolizumab in eight patients with ABPA reported since 2018. Treatment with mepolizumab resulted in the resolution of clinical symptoms and radiological pathologies. The mean time to improvement was calculated at 4.78 ± 4 months. One patient (Matsumoto et al.) responded to mepolizumab in as soon as two weeks (a rapid improvement) [12]. As we discussed, the goal of therapy in ABPA is a 25%-50% reduction in total IgE level, but among eight patients, only four patients reached a significant reduction in total IgE level after mepolizumab therapy. Therefore, the response to mepolizumab in ABPA is not associated with a reduction in total IgE level. Also, the total IgE level in two patients was <200 IU/mL, which may be considered in the reference range (i.e., 2-214 IU/mL); this evidence suggests that low total IgE level does not restrict mepolizumab therapy in ABPA [24].

In severe eosinophilic asthma, patients with a blood eosinophil count ≥ 150/mm^3^ at baseline or ≥300/mm^3^ during the previous year are assumed to have better responses to mepolizumab therapy [25]. In the current study, the mean eosinophil count before mepolizumab therapy was calculated at 1,990 ± 1,800/mm^3^, with the lowest number of 276/mm^3^. Eosinophil cell-death-mediated degranulation (eosinophil ETosis [EETosis]) is a programmed cell death pathway and considered to play an essential role in the mucus plug formation. For the first time in 2004, neutrophil extracellular traps (NETs) were described [26]. Subsequently, NETosis was defined as the novel cell death process, which mediates degranulation of neutrophils and the release of filamentous chromatin structures. NETs contain different antimicrobial molecules, including histones, elastase, myeloperoxidase, cathepsin, and lactoferrin, which immobilize and kill microorganisms. EETosis is a part of the innate immune system and was first described as a cytolysis mechanism that is involved in eosinophilic inflammatory diseases. Excess production of extracellular traps could be pathologic. For instance, significant numbers of NETs were detected in thick airway fluids from CF patients; treatment with inhaled recombinant human DNase improved lung function in these patients, indicating the role of NETs in the pathogenesis of viscous mucus formation. In addition, copious eosinophil extracellular cell death (EETs) was detected in bronchial mucus plugs of ABPA patients that were reduced after glucocorticoid treatment. The tremendous effects of mepolizumab in eight ABPA patients and the resolution of mucus plugs propose an essential role for EETosis in mucus plug formation of patients with ABPA.

Moreover, in patients with severe eosinophilic asthma treated with subcutaneous mepolizumab 100 mg every four weeks, at week 32, the mean FEV1 after bronchodilation increased to 138 mL compared to the placebo group; while according to our literature review, FEV1 improved by a mean of 375 mL after treatment with mepolizumab [10].

The most common reported adverse effects of mepolizumab include nasopharyngitis, headache, upper respiratory tract infection, sinusitis, bronchitis, oropharyngeal pain, and infection-site reaction; none of the mentioned adverse events were reported in above mentioned eight patients [10].

Limitations

Our study was limited in that we reviewed only eight cases. A randomized, double-blind clinical trial needs to be conducted to evaluate the efficacy of therapy with mepolizumab in ABPA and explore any adverse effects.

## Conclusions

Mepolizumab is a monoclonal antibody against IL-5 used for the treatment of severe eosinophilic asthma. We reviewed eight patients of ABPA treated successfully with mepolizumab. Treatment with mepolizumab was not restricted to the total IgE level, the issue that makes limited use of omalizumab in ABPA. Also, mepolizumab therapy improved FEV1, radiological findings, and quality of life. Mepolizumab can be considered as a glucocorticoid-sparing agent and a safe treatment in ABPA. Successful treatment of ABPA patients with mepolizumab suggests an essential role for EETosis in the pathology of ABPA, including mucus plug formation. Of note, double-blind placebo-controlled studies are required to establish the efficacy and safety of mepolizumab therapy for ABPA.
